# Unexpected abnormal coagulation test results in a 2-year-old child: A case report

**DOI:** 10.11613/BM.2020.011002

**Published:** 2019-12-15

**Authors:** Patricija Banković Radovanović, Tanja Živković Mikulčić, Jasmina Simović Medica

**Affiliations:** Department of transfusion medicine, General Hospital Pula, Pula, Croatia

**Keywords:** case report, preanalytical phase, sample collection, coagulation

## Abstract

Rejection of the sample with repeated blood withdrawal is always an unwanted consequence of sample nonconformity and preanalytical errors, especially in the most vulnerable population – children. Here is presented a case with unexpected abnormal coagulation test results in a 2-year-old child with no previously documented coagulation disorder. Child is planned for tympanostomy tubes removal under the anaesthesia driven procedure, and preoperative coagulation tests revealed prolonged prothrombin time, activated partial thromboplastin time and thrombin time, with fibrinogen and antithrombin within reference intervals. From the anamnestic and clinical data, congenital coagulation disorder was excluded, and with further investigation, sample mismatch, clot presence and accidental ingestion of oral anticoagulant, heparin contamination or vitamin K deficiency were excluded too. Due to suspected EDTA carryover during blood sampling another sample was taken the same day and all tests were performed again. The results for all tests were within reference intervals confirming EDTA effect on falsely prolongation of the coagulation times in the first sample. This case can serve as alert to avoid unnecessary loss in terms of blood withdrawal repetitions and discomfort of the patients and their relatives, tests repeating, prolonging medical procedures, and probably delaying diagnosis or proper medical treatment. It is the responsibility of the laboratory specialists to continuously educate laboratory staff and other phlebotomists on the correct blood collection as well as on its importance for the patient’s safety.

## Introduction

Preanalytical phase is still a major source of laboratory errors as well as major concern for laboratory specialists. Sample nonconformities, regardless being quality or quantity related, have a great impact on the patient results which, in consequence, might lead to several unwanted clinical outcomes compromising patient’s safety (*1*). In this regard, quality of coagulation samples is critical since lots of actions taken during phlebotomy may impair the reliability of these results (*2*). Besides the standard criteria for an appropriate and safe venepuncture (proper patient identification, appropriate devices as well as use of the correct technique), this especially refers to the type of the anticoagulant used, duration of venous stasis, order of draw as well as appropriateness of filling and mixing of the primary collection tube (*3*, *4*). Samples nonconformity cause rejection of the samples and repeated blood withdrawal which is an unwanted consequence of the preanalytical error especially for the most vulnerable population – children (*5*). Here is presented a case with unexpected abnormal coagulation tests results in a 2-year-old child with no previously documented coagulation disorder. Informed consent for publication of this article was obtained from the patient’s mother.

## Laboratory analyses and case story

Department of transfusion medicine received a sample of a 2-year-old child for coagulation testing. Due to persistent and recurrent ears inflammation (original diagnosis: *Otitis media acuta suppurativa recidivans bilateralis*) child has undergone the procedure called myringotomy 1.5 years ago; at the present time, the inserted tympanostomy tubes are planned to be removed under anaesthesia driven procedure, therefore, preoperative coagulation tests have been ordered by paediatrician. Coagulation tests were performed on the Siemens BCS XP System (Siemens Healthineers AG, Munich, Germany) with following reagents: Dade Innovin for prothrombin time (PT), Dade Actin FS for activated partial thromboplastin time (aPTT), Thromboclotin for thrombin time (TT), Multifibren U for fibrinogen (FBG) and Berichrom AT III for antithrombin (AT) determination. Results revealed prolonged PT, aPTT and TT with FBG and AT within reference intervals (Table 1, sample No. 3). The prolonged results were unexpected since this child has been tested before the myringotomy and adenoidectomy, 18 and 12 months ago, respectively (Table 1, sample No. 1 and 2), not showing any sign of coagulation disorder, neither in clinic nor laboratory findings. In order to unravel this, several points of investigation were elaborated.

**Table 1:** The results of all coagulation tests performed at different time points

**Sample**	**Time of sampling**	**PT (%)**	**APTT (s)**	**APTT (ratio)**	**FBG (g/L)**	**AT (%)**	**TT (s)**
No. 1	18 months ago	106	25	0.9	2.3	96	14
No. 2	12 months ago	113	22	0.8	3.1	118	13
No. 3	Present time (10:57 am)	43	52	1.9	2.3	111	34
No. 4	Present time (15:29 pm)	112	24	0.9	3.1	119	15
**Reference interval**		80 - 123	22 - 33	0.8 - 1.2	1.8 - 3.5	79 - 112	13 - 19
PT – prothrombin time. APTT – activated partial thromboplastin time. FBG – fibrinogen. AT – antithrombin. TT – thrombin time.							

## Further investigation

Since coagulation results were unexpectedly prolonged and not in line with the patient clinical data, we re-inspected the sample for clots presence due to the possible poor blood collection technique or the inappropriate mixing with the anticoagulant, which might result in consumption of coagulation factors. Even though all samples are regularly inspected after centrifugation and prior to testing by the visual observation of the plasma appearance as well as the appearance of the contact layer between sedimented cells and plasma, the sample with suspicious results was looked up again by using the wooden stick to fish out the presumed clot. No clot was detected so consumption of coagulation factor during the clot formation and consequently false prolonged results were excluded.

The next step was checking if the sample belongs to the patient. Since this patient has medical history in our hospital, his blood type was known, being A positive. We tested the sample for blood type, and it resulted A positive. Unfortunately, since this blood type could be found in 1/3 of the Caucasian, we did not confirm with certainty that the sample belongs to this patient (*6*).

We got into the contact with the phlebotomist to establish conditions under which phlebotomy was performed. Phlebotomist stated that this child was the first patient that morning, declining the possibility for sample mismatch. When phlebotomist was asked about the order of draw, she was not able to give the strait answer; she was confused, which raised the suspicion that this step was not performed correctly. Apart from the coagulation tests, samples were taken for complete blood count test as well as for erythrocyte sedimentation rate the same day. We suspected that ethylenediaminetetraacetic acid (EDTA) tube was collected prior to the coagulation tube which resulted in cross-contamination of the coagulation sample with EDTA.

We informed the child’s mother that a new sample had to be taken due to suspicious results. However, since there still was the slight possibility for accidentally ingestion of the oral anticoagulant, we had to inquire that too. The child was often in the proximity of the grandfather who takes some medications. However, after we interviewed mother, we found out that no one of the household takes anticoagulant therapy which makes this scenario not probable.

Heparin contamination as possible cause of such results was also excluded since no tube with heparin as anticoagulants were used and child was not under heparin therapy. Vitamin K deficiency due to malnutrition could have altered PT and APTT, however, since the child did not manifest symptoms of that deficiency: haematoma, purpura, nose bleedings, anaemia, *etc.*, vitamin K deficiency was highly unlikely hence discarded as possible cause of prolonged coagulation tests results. Furthermore, prolonged TT is not associated with vitamin K deficiency anyhow.

Remaining the only possible cause of the presented results, contamination with EDTA was explored through the repeated yet controlled procedure. The second sample was taken for coagulation tests by the ssame phlebotomist the same day and the same tests were performed. The results of all coagulation tests were within reference intervals (Table 1, sample No. 4) ruling out coagulation disorder and making previous contamination with EDTA as the most possible explanation.

## What happened?

We presented here a case with false prolonged coagulation tests results caused by EDTA contamination due to non-compliance to the standards for sample collection. This was confirmed after excluding several potential sources of false test results as well as repeating of blood withdrawal which consequently showed results within reference intervals. However, the magnitude of these prolongations is somehow larger than it would be expected for EDTA cross-contamination due to the incorrect order of draw. These prolongations rather resemble to the treatment with the oral anticoagulant, which, however, is excluded from the scenario. What we think could have happened is a matter that we have experienced several times with phlebotomists from other departments which are not familiar with the coagulation testing. Namely, when coagulation tubes are undefiled, these phlebotomists fill coagulation tubes with additional blood from other tubes. This is more likely to happen in this case as it may explain almost two-fold prolongation of the coagulation times for several tests. We think that contamination due to EDTA overflow was the cause of the false coagulation results.

## Discussion

Ethylenediaminetetraacetic acid is a chelating agent, binding divalent cations, like calcium, which is essential for the clotting mechanism (*7*). This makes EDTA suitable to be used as an anticoagulant. However, due to its chelating properties to continue with the calcium ions binding even if more calcium is added, like in coagulation tests, EDTA is not suitable as anticoagulant in samples assigned for coagulation testing (*8*). As such is used sodium citrate, which also chelates calcium, but its effect is easily reversible by addition of calcium ions (*7*).

Since the selection of proper anticoagulant is critical for coagulation tests and their reliability, the proper adherence to the current recommendation in blood collection is of utmost importance, considering appropriate collection, handling and processing of samples. Non-compliance to these recommendations will lead to inappropriate test results. Nowadays, coagulation analysers have reached highly accurate performance characteristics and, providing proper internal quality control as well as external quality assurance measures are performed, analytical errors within haemostasis testing are minimal (*3*). As opposite, preanalytical variables still represent prowling threat to the results accuracy, hence, patient safety.

Although approved Clinical and laboratory standards institute (CLSI) guidelines for phlebotomy procedure exist for years, as well as compatible national guidelines, there is still lack of adherence to them (*9*-*12*). An example of deviation from the CLSI and national guidelines for phlebotomy is presented in this case, relating to the incorrect order of draw or transfer of blood from one tube to another, or possibly both.

Several authors performed researches about the effect of the EDTA carryover during the phlebotomy. In the study about effect of the incorrect procedures during phlebotomy, Lima-Oliveira *et al*. showed 38% lower calcium concentration in a blood gas syringe taken after the tube with EDTA with conclusion that EDTA carryover occurred (*13*). Few years later the same group of authors investigated the impact on the coagulation tests in citrate samples contaminated with EDTA (*14*). They found that contamination of citrated blood with as much as 29% of EDTA blood generated a significant bias in results of routine clotting assays (PT and APTT) while fibrinogen was more robust, with significant bias at 43% EDTA contamination. According to these results authors concluded that the minimum cross-contamination of EDTA that may occur during collection of coagulation samples with evacuated tube systems is unlikely to generate any significant bias in results of APTT, PT, and fibrinogen. Other studies were in line with these conclusions stating that the order of draw, if the sampling performed correctly, has negligible effect on sample quality and that no significant influence on the results occurred due to (if any) contamination (*15*-*19*). This is also the opinion of the European Federation for Clinical Chemistry and Laboratory Medicine (EFLM) Working Group for the Preanalytical Phase (WG-PRE). However, since a significant frequency of sample contamination does still occur if order of draw is not followed during blood collection, they recommend continuing with the correct order of draw for venous blood collection (*20*).

Cadamuro *et al*. found that a simulated carryover of 1 µL of K_3_EDTA blood reduced calcium by 13% (*21*). According to Cadamuro *et al.*, EDTA whole blood carryover is highly unlikely, and, even if it occurs, it would be needed a larger volume than 10 µL to alter test results. Nevertheless, most of these studies did not consider the specific feature of the coagulation sample. Since chelating effect of EDTA on calcium is irreversible, that would mean not only 13% less calcium in the sample but also less calcium which is usually added to the sample for clotting tests initiation and sequestrated by EDTA. Due to that fact coagulation tests becomes partly or completely inhibited. However, would this calcium reduction have led to the almost two-fold prolongation of the coagulation times has not been studied here, hence, cannot be stated as such nor it can be stated what degree of calcium reduction in our case was. The answer on that issue could be found in a study performed by d’ Audigier *et al.* (*22*). They performed repeated sampling for those samples which exhibited low calcium level (“Ca 0” samples) and performed coagulation tests in the new samples: PT, APTT, TT, FBG, factors V (FV), VII (FVII) and X (FX), comparing them with the same tests obtained in “Ca 0” samples. They found significant decrease of the fibrinogen concentration and FV coagulant activity as well as prolongation of the PT, APTT and TT coagulation times. Their conclusion was that improper anticoagulant present in a citrated tube could result from decanting part of an EDTA sample to an insufficiently filled citrated tube leading to an utmost imbalance of the citrate-calcium ratio.

Considering that as well as the fact that our results were consistent with these findings, we can agree with Cadamuro and d’ Audigier that contamination of sample with preloaded pure EDTA solution could be possible explanation in presented case since we witnessed many times to this matter as stated above.

In line with its way of acting on calcium, by chelating it and making it absent in the coagulation process, EDTA inevitably has effect on coagulation tests results by prolonging time for the clot formation. Unfortunately, it is not possible to recognize EDTA contamination by observation of the sample after centrifugation; this could be suspected only *post festum*, when unusual or unexpected results appear. At that point it is important to obtain accurate data about conditions of the performed phlebotomy which can be very challenging especially if the sample was taken outside the laboratory where the tests were performed. Phlebotomist could be confused or even embarrassed reacting defensively and hiding some facts that could be important for the conclusion and possible solution, making their testimony doubtful. In such situation the only possible way to confirm EDTA contamination in the first sample is to perform blood collection again expecting results within reference intervals in the new sample. However, this is easier to do when it concerns adults; when a child is a patient, lots of regarding issues makes that more difficult. Understandably, parents are often suspicious and worried, child fears the blood drawing again and every excessive blood sampling is unwanted for this vulnerable population. Moreover, if anamnestic data are not known, child is referred to the clinic for further investigation which would delay the planned operation. That obliges everyone included in this process trying to avoid this situation by doing it right the first time.

To conclude, due to the poor cooperation of the phlebotomist, we cannot state with certainty what happened in this case, but what is certain is that non-compliance to the current recommendations in blood collection or severe neglect had occurred which resulted with the EDTA contamination and consequently false prolongation of coagulation times. Unfortunately, preanalytical phase, including sample collection, is often outside of control of the laboratory, as it can be seen in presented case. This put laboratory specialist to the mission for continuous education of the phlebotomists.

What YOU should/can do in your laboratory to prevent such errors

1. Education of laboratory staff and phlebotomist about correct blood collection and its impact on laboratory results should be continuous task of laboratory specialists.
2. Laboratories should provide phlebotomists with the leaflets with the specific instructions for all issues in the preanalytical phase.
3. Continuous communication between laboratory and phlebotomist of provenance outside the laboratory should be adopted in a way of advising phlebotomists on preanalytical issues in various situations. Supportive and helpful approach of laboratory staff to the phlebotomists is a valuable step to achieve better mutual trust and successful collaboration for higher goal: the patient safety.
4. To avoid unnecessary loss, everyone involved in the patient care should embrace the following quality principle: Do it right the first time.
